# Block of Voltage-Gated Sodium Channels by Atomoxetine in a State- and Use-dependent Manner

**DOI:** 10.3389/fphar.2021.622489

**Published:** 2021-02-25

**Authors:** Karl Josef Föhr, Ariadni Nastos, Michael Fauler, Thomas Zimmer, Bettina Jungwirth, David Alexander Christian Messerer

**Affiliations:** ^1^ Department of Anesthesiology and Intensive Care Medicine, University Hospital Ulm, Ulm, Germany; ^2^ Department of General Physiology, Ulm University, Ulm, Germany; ^3^ Institute of Physiology, University Hospital Jena, Jena, Germany

**Keywords:** atomoxetine, hNav1.5, cardiotoxicity, antiarrhythmic, patch clamp, attention-deficit/hyperactivity disorder

## Abstract

Atomoxetine, a neuroactive drug, is approved for the treatment of attention-deficit/hyperactivity disorder (ADHD). It is primarily known as a high affinity blocker of the noradrenaline transporter, whereby its application leads to an increased level of the corresponding neurotransmitter in different brain regions. However, the concentrations used to obtain clinical effects are much higher than those which are required to block the transporter system. Thus, off-target effects are likely to occur. In this way, we previously identified atomoxetine as blocker of NMDA receptors. As many psychotropic drugs give rise to sudden death of cardiac origin, we now tested the hypothesis whether atomoxetine also interacts with voltage-gated sodium channels of heart muscle type in clinically relevant concentrations. Electrophysiological experiments were performed by means of the patch-clamp technique at human heart muscle sodium channels (hNav1.5) heterogeneously expressed in human embryonic kidney cells. Atomoxetine inhibited sodium channels in a state- and use-dependent manner. Atomoxetine had only a weak affinity for the resting state of the hNav1.5 (Kr: ∼ 120 µM). The efficacy of atomoxetine strongly increased with membrane depolarization, indicating that the inactivated state is an important target. A hallmark of this drug was its slow interaction. By use of different experimental settings, we concluded that the interaction occurs with the slow inactivated state as well as by slow kinetics with the fast-inactivated state. Half-maximal effective concentrations (2–3 µM) were well within the concentration range found in plasma of treated patients. Atomoxetine also interacted with the open channel. However, the interaction was not fast enough to accelerate the time constant of fast inactivation. Nevertheless, when using the inactivation-deficient hNav1.5_I408W_L409C_A410W mutant, we found that the persistent late current was blocked half maximal at about 3 µM atomoxetine. The interaction most probably occurred via the local anesthetic binding site. Atomoxetine inhibited sodium channels at a similar concentration as it is used for the treatment of ADHD. Due to its slow interaction and by inhibiting the late current, it potentially exerts antiarrhythmic properties.

## 1 Introduction

Atomoxetine belongs to the group of norepinephrine transporter (NET) inhibitors. It is the first nonstimulant compound licensed for the treatment of attention-deficit/hyperactivity disorder (ADHD) (Peterson et al., 2008). Increased intrasynaptic norepinephrine (NE) levels are detectable shortly after oral application of low doses of atomoxetine (Takano et al., 2009). However, therapeutic effects occur not until 2–3 weeks after application of much higher drug doses (Kratochvil et al., 2002; Hiemke et al., 2011). Thus, when therapeutic concentrations of atomoxetine are applied, the interaction of atomoxetine may no longer be restricted to noradrenaline transporter. Indeed, using clinically relevant concentrations of atomoxetine we and others found further interactions with different ion channels (Scherer et al., 2009; Ludolph et al., 2010). A similar behaviour is also known for other neuroactive drugs which in therapeutic relevant concentrations do not solely interact with their main target but also with ion channels, predominantly with ligand gated ion channels (Lenkey et al., 2006). In this study, we investigated whether atomoxetine also interacts with voltage-gated sodium channels (VGSCs).

### 1.1 Voltage-Gated Sodium Channels

VGSCs are composed from one out of 9 different pore forming α-subunits which are associated with no, one or two out of 4 auxiliary ß-subunits (Catterall et al., 2005; Patino and Isom, 2010). The α-subunits are designated as Na_v_1.1 to Na_v_1.9 according to their phylogeny. Other diversity arises from alternative splicing of mRNA and post-translational modifications. The distribution of the different subunits is tissue specific. Na_v_1.4 and Na_v_1.5 are predominantly found in skeletal and heart muscle cells, whereas Na_v_1.1 to Na_v_1.3 or Na_v_1.8 occur in neurons. According to pharmacological parameters the individual channels are classified as TTX-sensitive or TTX-resistant, depending on the concentration of tetrodotoxin (TTX) which is required for their blockage. Further classifications are based on different subunit specific electrophysiological properties (Theile and Cummins, 2011).

### 1.2 Voltage-Gated Sodium Channels and Psychotropic Drugs

The chemical structure of atomoxetine closely resembles that of fluoxetine which is commonly used as a selective serotonin reuptake inhibitor. From this point of view, it is not surprising that both drugs block NMDA receptors (Szasz et al., 2007; Ludolph et al., 2010; Barygin et al., 2017). Beyond this, fluoxetine is also known to block VGSCs (Lenkey et al., 2006; Poulin et al., 2014). Therefore, we investigated if and how atomoxetine interacts with VGSCs. For this purpose, we chose the cardiac subtype (hNa_v_1.5) as many psychotropic drugs have the potential to enhance the risk for a sudden death of cardiac origin (Timour et al., 2012).

## 2 Methods

### 2.1 Cell Culture

The tsA201 cell line is a derivative of the human embryonic kidney cell line HEK-293 (ATCC#CRL1537, Merck KGaA, Darmstadt, Germany). tsA201 cells were cultured at 37°C in a humidified atmosphere at 95% air and 5% CO_2_ in MEM (minimum essential medium, Gibco, Eggenstein, Germany) supplemented with 50 U/ml penicillin, 50 μg/ml streptomycin (Gibco), 2 mM L-glutamine (Boehringer, Mannheim, Germany), and 10% fetal calf serum (Gibco). The cells were grown on polyornithine-coated culture dishes to 40% confluency and transfected using the TransFectin LipidReagent kit (Bio-Rad, München, Germany). The construction of the plasmid pTSV40G-hNa_v_1.5 encoding wild-type hNa_v_1.5 was described previously (Walzik et al., 2011). This plasmid allows for the simultaneous production of enhanced green fluorescent protein (EGFP) from a separate expression cassette, and thus for the selection of transfected cells. Mutant channels hNa_v_1.5_I408W_L409C_A410W (hNa_v_1.5_WCW) and hNa_v_1.5_I408W_L409C_A410W_F1760K (hNa_v_1.5_WCW_ F1760K) were obtained by respectively modified oligonucleotides and overlapping polymerase chain reaction (PCR). The PCR fragments were inserted into the pTSV40G-hNa_v_1.5 background using restriction sites Age/BsaBI (for WCW) and BstEII/SpeI (for F1760K), resulting in pTSV40G-hNa_v_1.5_WCW and pTSV40G-hNa_v_1.5_WCW_F1760K.

### 2.2 Electrophysiology

Electrophysiological experiments were performed as previously described (Ludolph et al., 2010). Briefly, tsA201 cells were used for experiments 24–48 h after transfection. Membrane currents were recorded in the whole-cell recording mode using an EPC-9 amplifier and Patchmaster software (v2x73; HEKA, Lambrecht, Germany (Hamill et al., 1981)). Before recording, cells were rinsed twice with an extracellular standard solution containing (in mM): 140 NaCl, 5 KCl, 1.5 CaCl_2_, 10 glucose and 12 HEPES; adjusted to a final pH of 7.3. Patch pipettes were drawn from borosilicate glass with tip resistances of about 2 MΩ when filled with (in mM): 125 CsF, 10 NaF, 1 MgCl_2_, 10 EGTA, 10 HEPES; adjusted to a final pH of 7.2. To improve sealing, tips were briefly dipped into 2% dimethylsilane dissolved in dichloromethane. Unless otherwise stated, cells were held at a holding potential of −140 mV from which channel activation was elicited by brief depolarizing pulses to −20 mV of 5 ms duration. To minimize voltage errors, the series resistance was compensated up to 80%. Cells with currents larger than 6 nA were excluded from evaluation. Specific protocols are illustrated in figure legends where appropriate.

### 2.3 Drug Application

The medium in the dish (1.5 ml) was continuously exchanged using a "global" bath perfusion with the inflow set to 4.5 ml/min and the outflow removing any excess fluid. Reagents were applied locally to the cells by the L/M-SPS-8 superfusion system (List, Darmstadt, Germany). Switching between the 8 channels of the superfusion system was controlled by magnetic valves. The local inlet (tip of an eight-barrelled pipette) was positioned at a distance of 50–100 μm upstream and the local outlet at about 300 μm downstream of the patch pipette. A constant flow rate of control and test solutions (1 ml/min) was achieved by means of a pressure control system (MPCU-3, Lorenz, Göttingen, Germany). The time of solution exchange was estimated from the changes in the liquid junction potential to be about 1 ms. If not otherwise stated, drugs were preapplied for 20 s before starting the experiments.

### 2.4 Chemicals

Dulbecco's modified Eagle medium, penicillin/streptomycin, and glutamine were purchased from Gibco. Trypsin was obtained from Biochrom AG, Berlin, Germany. DNAse 1 was obtained from Invitrogen, Carlsbad, Germany; fetal calf serum was obtained from HyClone, Perbio Science, Bonn, Germany. Poly-L-ornithine was purchased from Sigma-Aldrich, Schnelldorf, Germany. Atomoxetine and all other chemicals were obtained from Sigma-Aldrich Chemie GmbH, Steinheim, Germany.

### 2.5 Data Analysis and Statistics

#### 2.5.1 Concentration-Inhibition Curves

Concentration-inhibition curves for the estimation of K_r_ (IC_50_ at −140 mV; Figure 2) or K_app_ (Figure 6) were fitted to the Hill equation

$$\frac{{\text{I}}_{\text{D}}}{{\text{I}}_{\text{C}}}=\;\frac{1}{1+{\left(\frac{\left[\text{D}\right]}{{\text{IC}}_{50}}\right)}^{\text{n}}}$$ (1)

I_D_ and I_C_ are the current amplitudes in the presence and absence of the drug (D). [D] is the concentration of the drug. IC_50_ represents the concentration of the blocker that causes 50% inhibition and n is the Hill coefficient.

#### 2.5.2 Voltage Dependent Behaviour

Voltage dependence of activation was calculated in two steps: First, changes in driving force owing to the different test potentials were considered by calculating the conductance g according to

$$\text{g }=\;\frac{\text{I}}{\text{V}-{\text{E}}_{\text{Na}}}$$ (2)

Thereafter, normalized data were fitted with a Boltzmann equation of the form:

$$\frac{\text{g}}{{\text{g}}_{\text{max}}}=\;\frac{1}{1+{\text{e}}^{\left(\frac{{\text{V}}_{50}\;-\text{ V}}{\text{k}}\right)}}$$ (3)

Voltage dependence of fast inactivation were fit using a Boltzmann equation of the form:

$$\frac{\text{I}}{{\text{I}}_{\text{max}}}=\;\frac{1}{1+\;{\text{e}}^{\left(\frac{\text{V }-\;{\text{V}}_{50}}{\text{k}}\right)}}$$ (4)

In case of slow inactivation Eq. 4 was extended by a second term and the additional parameter (S) which considers the steady-state level of the incomplete inactivation.

$$\frac{\text{I}}{{\text{I}}_{\text{max}}}=\left(1-\text{S}\right)\left(\frac{{\text{a}}_{1}}{1+{\text{ e}}^{\left(\frac{\text{V}-\text{ V}50_{1}}{{\text{k}}_{1}}\right)}}+\;\frac{{\text{a}}_{2}}{1+{\text{ e}}^{\left(\frac{\text{V}-\text{ V}50_{2}}{{\text{k}}_{2}}\right)}}\right)+\text{ S}$$ (5)

Abbreviations used for voltage-dependent parameters: V and V_50i_ are the actual membrane potentials and the potentials at which half maximal current (I)/conductance (g) occur, respectively. E_Na_ indicates the reversal potential which was experimentally determined for each cell. The slope factors are given by k_i_ and the relative amount of the individual terms are represented by a_i_. Constraint throughout: $\overset{\text{n}}{\underset{\text{i}=1}{\sum }}a_{\text{i}}=1$.

#### 2.5.3 Interaction With the Inactivated State

##### 2.5.3.1 Estimation of Apparent Binding Constants

The affinity to the inactivated state (K_i_) was calculated according to (Bean et al., 1983) as:

$$\frac{1}{{\text{K}}_{\text{app}}}=\;\frac{\text{h}}{{\text{K}}_{\text{r}}}+\;\frac{\left(1-\text{h}\right)}{{\text{K}}_{\text{i}}}$$ (6)

K_app_ is the apparent affinity estimated at a selected membrane potential at which the amount of non-inactivated channels is given by h (estimated from the corresponding inactivation curve). K_r_ is the affinity for the resting channel (120 µM).

##### 2.5.3.2 Time and Concentration Dependent Development of Block

The time constant (*τ*) of block development for the different concentrations of atomoxetine was estimated by single exponential fits of the form:

$$\frac{\text{I}}{{\text{I}}_{\text{max}}}\;=\text{ S}+{\text{ a}}_{1}{\text{*e}}^{\left(\frac{-\text{t}}{{\text{τ}}_{1}}\right)}\;$$ (7)

t denotes the time, a is the fraction of channels which is blocked, and S represents the residual current.

Association and dissociation rates:

Association (k_on_ in µM^−1^s^−1^) and dissociation rates (k_off_ in s^−1^) were estimated from the slope and the *y*-intercept of a linear regression where the inverse of the fast time constants (1/τ) was plotted vs. the drug concentration [D] (Kuo and Lu, 1997).

$$\frac{1}{\text{τ}}={\text{ k}}_{\text{off}}+{\text{k}}_{\text{on}}\text{*}\left[\text{D}\right]$$ (8)

The affinity for the inactivated state (K_i_) was calculated according to:

$${\text{K}}_{\text{i}}=\;\frac{{\text{k}}_{\text{off}}}{{\text{k}}_{\text{on}}}$$ (9)

#### 2.5.4 Recovery From Inactivation

Current amplitudes were normalized to the maximum peak amplitude of I_Na_ in the absence and presence of atomoxetine. Recovery time constants were estimated from double or triple exponential fits according to:

$$\frac{\text{I}}{{\text{I}}_{\text{max}}}\;=\;1-{\text{ a}}_{1}{\text{*e}}^{\left(\frac{-\text{t}}{{\text{τ}}_{1}}\right)}\;-{\text{a}}_{2}{\text{*e}}^{\left(\frac{-\text{t}}{{\text{τ}}_{2}}\right)}-{\text{ a}}_{3}{\text{*e}}^{\left(\frac{-\text{t}}{{\text{τ}}_{3}}\right)}$$ (10)

Variable identifiers have the same meaning as in Eqs. 5, 7.

#### 2.5.5 Curve Fitting and Statistics

All curve fitting procedures were performed using SigmaPlot 10.0 (Sysstat, San Jose, California, United States).

Single exponential fits of original current traces were performed using fitmaster software (v2x73, Heka). If not directly stated by the presence of error bars, graphs show representative data from single cells. Average values from *N* = 5 cells are given as mean ± SD in the results section and in figure legends. Single comparisons of paired data were calculated with a two-sided Wilcoxon’s signed rank test (Matlab V9.0, MathWorks, Natick, Massachusetts, United States) and considered significant if *p* < 0.05.

## 3 Results

### 3.1 Atomoxetine Blocks Human Na_v_1.5 Channels

**FIGURE 1:**
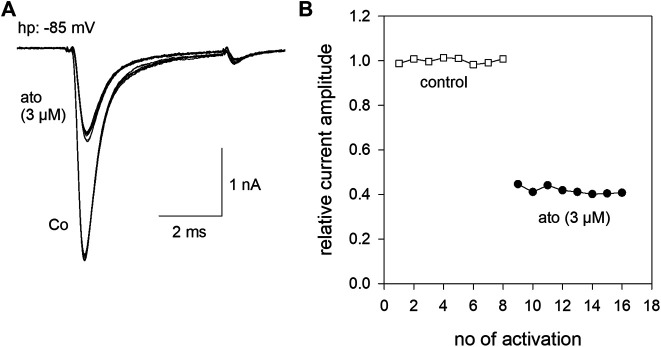
Inhibition of hNav1.5 by atomoxetine **(A)** Representative current traces obtained from infrequent channel activations every 30 s by step depolarization from a holding potential of −85 mV to −20 mV for 5 ms. Current amplitudes are almost stable during repeated activations in the absence (Co.) or presence of atomoxetine (ato). The overlay of 8 current traces is shown **(B)** Normalized current amplitudes of individual activations in the absence and presence of atomoxetine (3 µM).

All experiments were performed with hNa_v_1.5 wild-type or mutant channels, transiently transfected in tsA201 cells. Brief depolarizations for 5 ms to −20 mV from a holding potential close to half maximal inactivation (here −85 mV) resulted in typical fast inactivating inward currents. The size and shape of these currents did not change upon infrequent activations (every 30 s) when control solution was applied. However, when the perfusion was switched to 3 µM atomoxetine the current amplitude instantaneously dropped to a new value and stayed almost constant upon ongoing activations in the presence of atomoxetine (Figure 1).

### 3.2 Interaction With the Resting State

**FIGURE 2:**
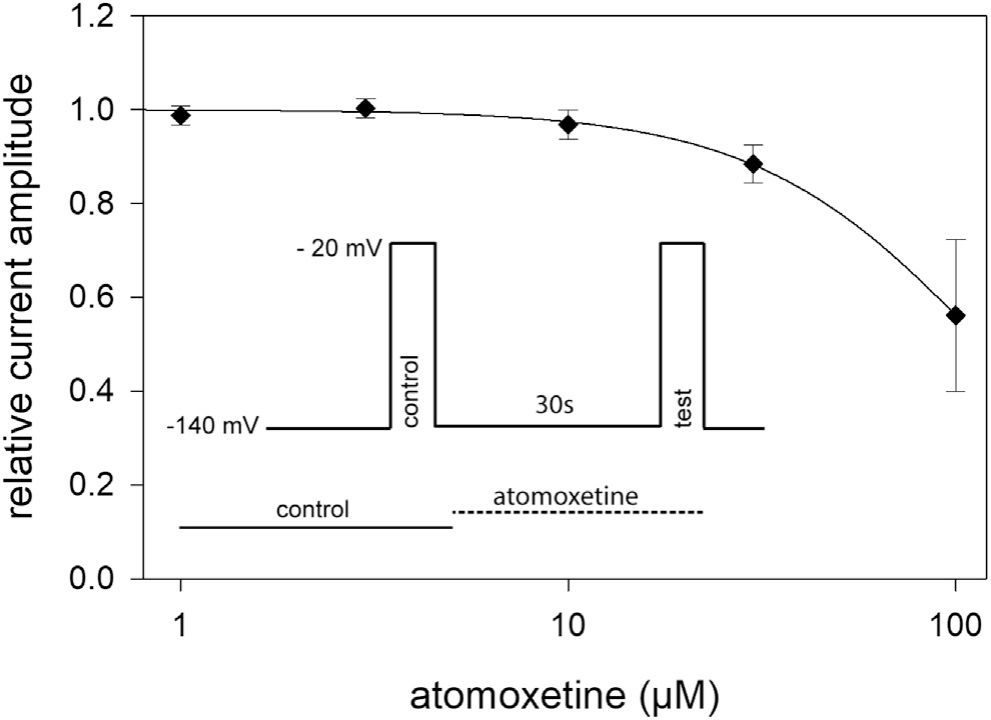
Tonic block of hNav1.5 channels by atomoxetine. Normalized current amplitudes obtained from the activation of hNav1.5 channels in the absence and presence of different concentrations of atomoxetine. Channels were activated from a holding potential of −140 mV. Current amplitudes in the presence of atomoxetine (preapplied for 30 s) were related to the respective control pulse (inset). Solid lines represent fits to Eq. 1. Half maximal effects were obtained at 119 ± 2.8 µM with a Hill-coefficient of 1.47 ± 0.06. *N* = 5.

In order to estimate tonic current inhibition, cells were held at a potential of −140 mV to ensure that almost all channels were in the resting state. To avoid an accumulation of drug effects, we did not successively increase the drug concentrations. Instead of this, we used a double pulse protocol, where the control value was re-estimated for each drug concentration. When the individual control values obtained from one cell varied by more than 10%, data were not considered further. From our preliminary experiments, we chose a drug preapplication time of 30 s before the test pulse was carried out in the presence of different concentrations of atomoxetine (1–100 μM, Figure 2, inset). Prominent current reductions were observed with concentrations equal to or higher than 30 µM. Altogether, we estimated a half maximal effective concentration of 119 ± 2.8 µM with a Hill coefficient of 1.47 ± 0.06 (Figure 2). Compared to the inhibition evoked by 3 µM atomoxetine at a potential of about half-maximal inactivation, the inhibitory potency of atomoxetine was much reduced when the holding potential was set to −140 mV, indicating that the interaction of atomoxetine with hNa_v_1.5 channels happens in a voltage or state-dependent manner.

### 3.3 Activation and Fast Inactivation

**FIGURE 3:**
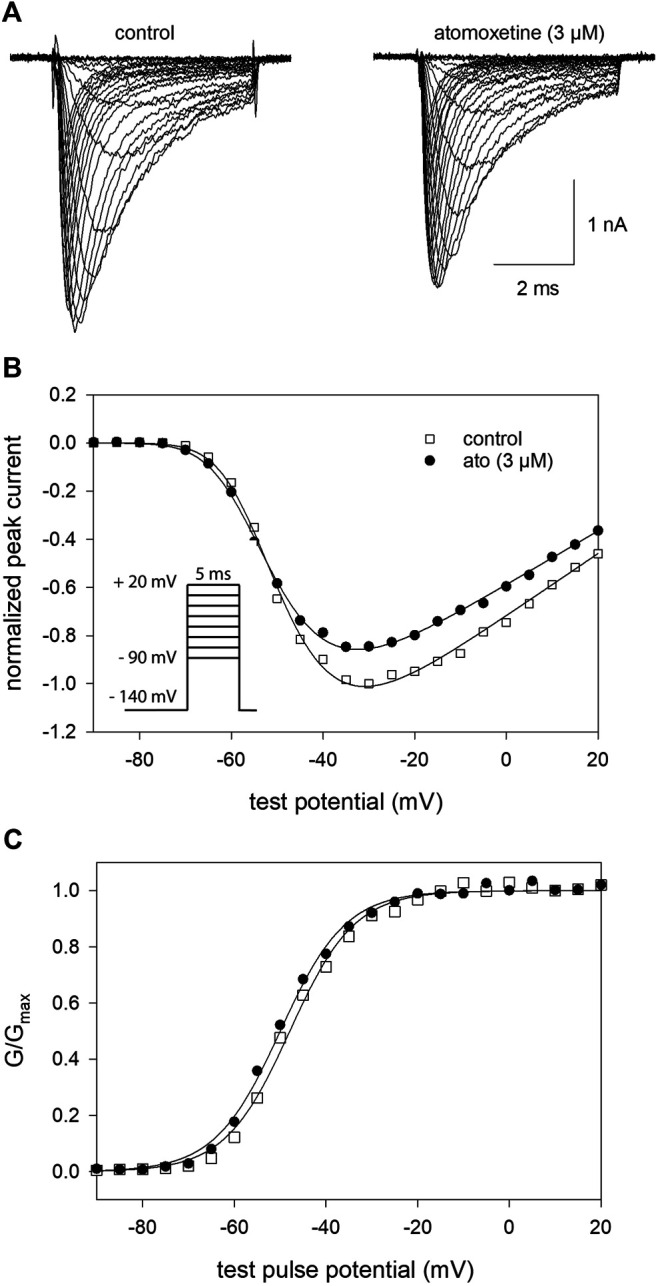
Voltage dependence of activation **(A)** Overlay of representative original current traces obtained from the activation of hNav1.5 channels in the absence (left) and presence of 3 µM atomoxetine (right). Cells were clamped at a holding potential of −140 mV from which sodium currents were evoked by 5 ms test pulses to potentials ranging from −90 to +20 mV (increment 5 mV; see inset in B) **(B)** Plot of normalized peak currents from the cell shown in A in the absence (open squares) and presence of 3 µM atomoxetine (filled circles) vs. test potential. Current amplitudes were normalized to the maximum of control which was reduced by 12.8 ± 2% in the presence of 3 µM atomoxetine **(C)** Plots of normalized peak conductance (G/G_max_) from the cell as shown in A in the absence and presence of atomoxetine as a function of the test pulse potential (symbols as in B). Solid lines are fits according to Eqs. 2, 3. Mean half maximal activation for control and in the presence of atomoxetine (3 µM) occurred at −52.6 ± 3.5 mV and −54.5 ± 3.9 mV, respectively, *N* = 5.

In a next step, we determined the voltage dependence of activation in the absence and presence of atomoxetine. To this end, depolarisations from a holding potential of −140 mV to different test pulse potentials (range: −90 to +20 mV in steps of 5 mV) were carried out. Original traces thereof are illustrated by Figure 3A. Atomoxetine (3 µM) exerts only a minor inhibitory effect on the maximal current amplitude (Figure 3B). From 5 independent investigations, we estimated a current reduction by 12.8 ± 2%. To analyse for a feasible shift of the potential dependent behaviour, we estimated midpoints of the activation curves in the absence and presence of atomoxetine using Eqs. 2, 3 (Figure 3C). Under control condition, the midpoints of the activation curves resided at −52.6 ± 3.5 mV with a slope of 6.6 ± 0.8 mV. The corresponding values in the presence of atomoxetine (3 µM) were −54.5 ± 3.9 mV with a slope of 6.6 ± 0.6 mV. When the concentration of atomoxetine was further increased to 10 μM, the midpoints of activation were further shifted to more negative values (−55.5 ± 4.1 mV; slope 6.9 ± 0.6 mV; not illustrated in Figure 3). Thus, activation of hNa_v_1.5 channels would even be facilitated in the presence of atomoxetine, indicating that channel activation is not a target for the inhibitory action of atomoxetine.

**FIGURE 4:**
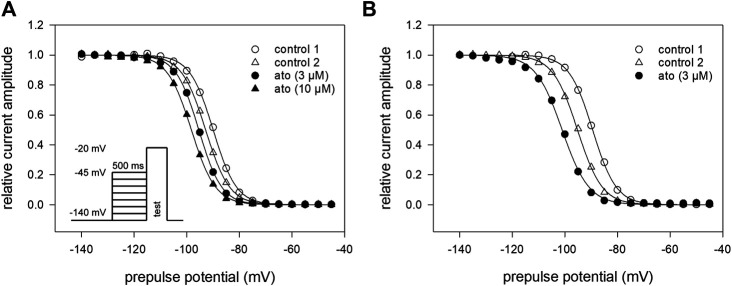
Voltage dependence of fast inactivation **(A)** Data of a representative measurement series of normalized peak currents in the absence and presence of atomoxetine are shown. Solid lines represent fits according to Eq. 4. Half maximal inactivation occurred for the first control at −88.1 mV ± 4.7 mV (range: −82.9 to −91.6 mV) with a slope of 4.6 ± 0.2 mV. The amount of the endogenous shift, estimated for each cell individually from the difference between the two controls, varied between 0.3 and 1.12 mV/min. Net drug-induced shift was +0.3 mV and −0.9 mV for 3 and 10 µM atomoxetine, respectively (*N* = 5) **(B)** Typical example for a cell with an extreme endogenous shift (2.24 mV/min), representative for two other cells. Atomoxetine (3 µM) does not provoke a relevant shift.

For establishing inactivation curves, the membrane potential was stepped to different prepulse potentials (from −140 to −45 mV; increment 5 mV) for 500 ms before channel activations were carried out by depolarizations to −20 mV. For estimation of inactivation parameters, the normalized peak currents were plotted vs. the prepulse potentials. Inactivation curves, characterized by inactivation midpoints and the curve slope were obtained by data fitting using Eq. 4. At control condition (control 1), mean half-maximal inactivation occurred at −88.1 ± 4.6 mV (range: −82.9–91.6 mV) with a mean slope of 4.6 ± 0.2 mV. After a second control, atomoxetine was applied at a concentration of 3 and 10 µM (Figure 4A) For final evaluation of drug effects, first the endogenous shift was estimated for each cell individually by the difference between the two controls. It varied in this experimental series between 0.33 and 1.12 mV/min. All in all, a net shift in positive direction by 0.3 mV and a negative shift by 0.9 mV were calculated for 3 and 10 µM atomoxetine, respectively. We used a cell-specific endogenous shift instead of a global shift, as the endogenous shift is highly variable between individual cells. Even though the endogenous shift might not behave perfectly linear during different repeats within one cell it is less variable than between individual cells. To illustrate this, we provide an extreme example with a large endogenous shift (2.24 mV/min) from another experimental series (Figure 4B). However, it is evident that the shift in the presence of atomoxetine (3 µM) is not stronger than between the preceding controls. A corresponding outcome was obtained from cells with a small endogenous shift (not shown). These observations indicate, that the amount of the endogenous shift has no impact on the drug-induced shift. Altogether, atomoxetine does not interact with fast inactivation as no relevant shift of inactivation mid points resulted.

### 3.4 Interaction With the Inactivated State: Kinetic Parameters

**FIGURE 5:**
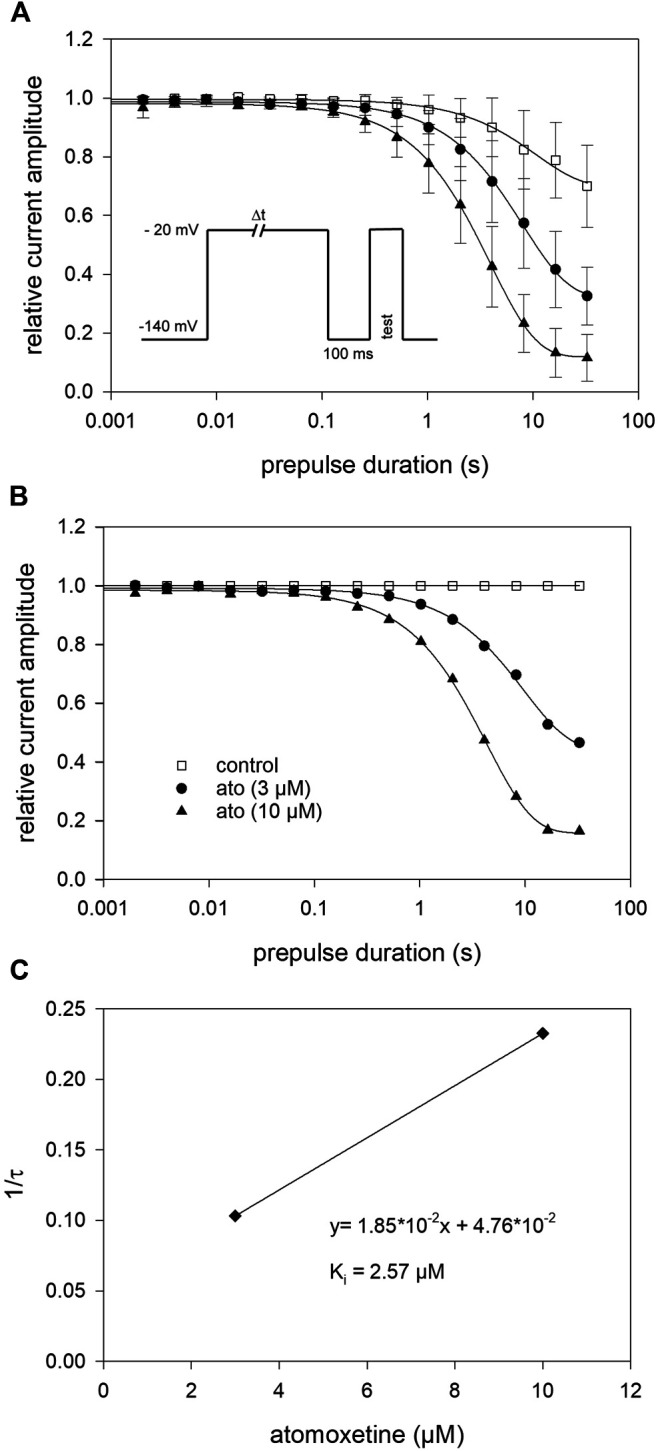
Block development **(A)** For estimation of open-state inactivation, the voltage of the conditioning prepulse was set to −20 mV. The pulse protocol (inset) was applied in intervals of 20 s. The peak currents of the test pulse were normalized to the peak amplitude of the prepulse and plotted vs. its duration. Solid lines represent fits of single exponential functions to data obtained for control (open squares) or different concentrations of atomoxetine **(B)** Data normalized with respect to control **(C)** The inverse of the time constants of individual cells were plotted vs. the concentration of atomoxetine. Association (k_on_) and dissociation rate constants (k_off_) were estimated from the slope and y-intercept of the linear fit to be 0.0185 µM^−1^s^−1^ and 0.047 s^−1^ (Eq. 8). The affinity to the inactivated state (Ki = 2.57 µM) was calculated according to Eq. 9. *N* = 5.

Due to the very small effect of atomoxetine on fast inactivation (500 ms), we did not test higher concentrations of atomoxetine here. Instead of this, we employed another double-pulse protocol in order to determine the time dependence of the interaction of atomoxetine with the inactivated state. The experimental scheme consisted of a conditioning prepulse to −20 mV for a variable duration (range: 0.002–32 s), a short recovery for 100 ms at −140 mV, followed by the test pulse to −20 mV (inset to Figure 5A). In the absence of atomoxetine, the control values became smaller at inactivation times lasting longer than 1 s and dropped to 70.0 ± 14.0% of its original value at the longest inactivation time tested. In the presence of atomoxetine (3 µM), the decline in the relative current amplitude started earlier with a much stronger final reduction (remaining current: 32.6 ± 9.8%). With the higher concentration of atomoxetine (10 µM), the inhibitory effect became even stronger (remaining current: 11.5 ± 8.0%, Figure 5A). For further evaluation, we normalized the data to control values and fitted the time course with single exponentials, giving time constants of 9.7 ± 0.49 s and 4.3 ± 0.1 s for 3 and 10 µM atomoxetine, respectively (Figure 5B). For estimation of rate constants the inverse of the time constants (1/τ) were plotted vs. the concentration of atomoxetine (Figure 5C). From the linear fit of this graph, we calculated a rate constant for association of 0.0185 µM^−1^s^−1^ and for dissociation of 0.047 s^−1^, resulting in a calculated K_i_ of 2.57 µM (Eq. 8 and Eq. 9).

### 3.5 Interaction With the Inactivated State: Steady-State Parameters

**FIGURE 6:**
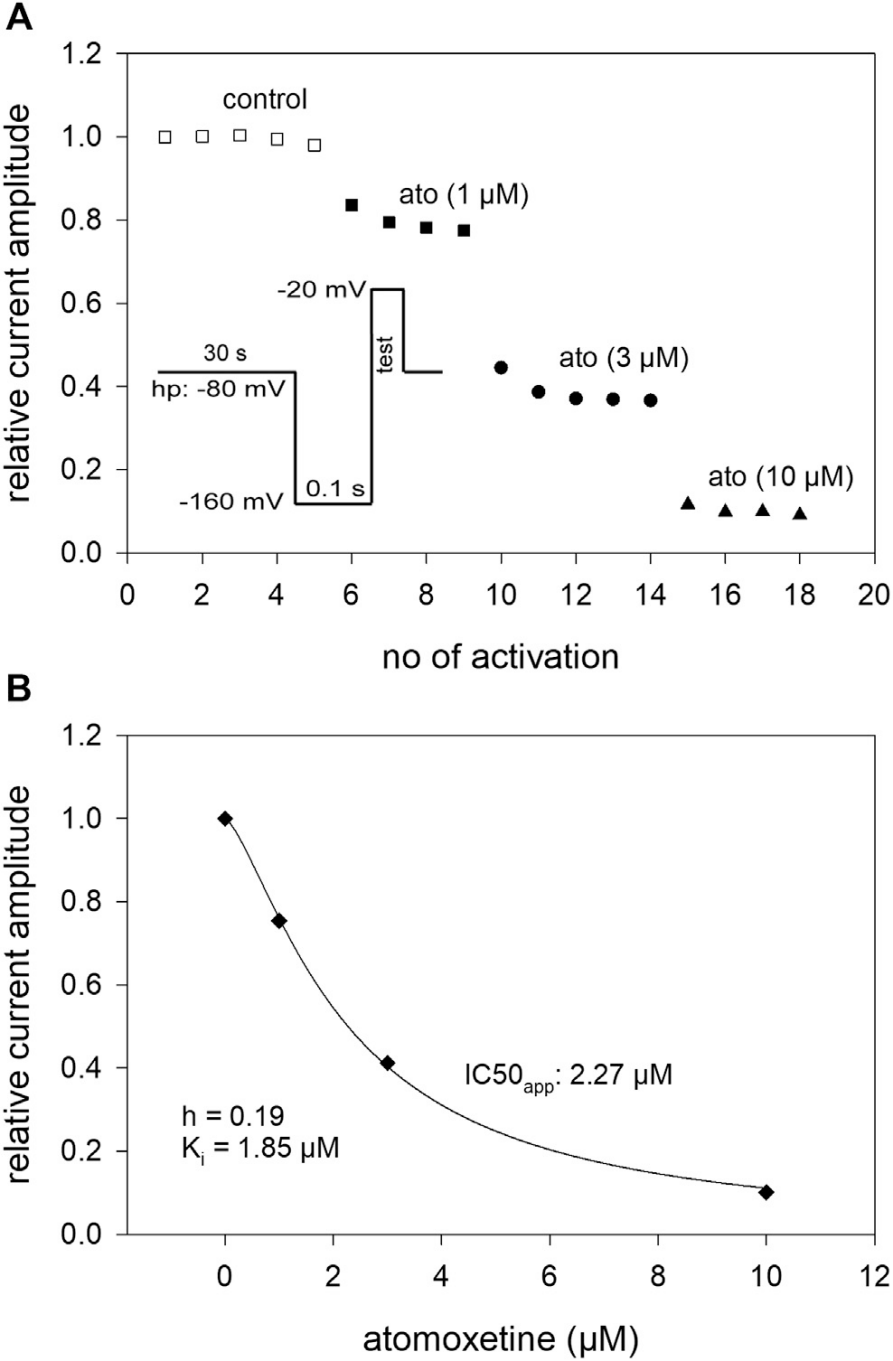
Interaction with the inactivated state: concentration-response relationship **(A)** The graph shows relative current amplitudes upon channel activations in the absence and presence of different concentrations of atomoxetine. Activations were carried out every 30 s using a holding potential at which the channels were partly inactivated (relative amount of available channels in this experiment: h = 0.81). Immediately before the activations a short recovery (100 m s) at −160 mV was intercalated (inset). Activations were repeated at each condition until a near stable current amplitude was achieved. **(B)** The apparent IC_50_ (IC_50app_) was calculated from the concentration-dependent decline of the current amplitudes. The affinity to the inactivated state was calculated considering the current amount of available channels according to Eq. 6. Altogether an affinity for the inactivated state (K_i_) of 1.59 ± 0.45 µM resulted, *N* = 5.

Due to the slow interaction of atomoxetine with the hNa_v_1.5 channel, we used long drug application times and infrequent activations (once every 30 s) for the establishment of a concentration relationship. Activations were repeated several times at each concentration to achieve steady-state condition (Figure 6A). The holding potential was chosen individually for each cell to be around half maximal inactivation according to a preceding estimation of availability. Before the test pulse a short recovery (100 ms at −160 mV) was carried out (inset to Figure 6A). From the concentration relationship apparent half maximal effective concentrations (K_app_) were calculated using Eq. 1 (Figure 6B). The affinity for the inactivated channel was then calculated according to Eq. 6 which considers the varying amount of inactivated channels in individual experiments. Altogether, we estimated an affinity of 1.59 ± 0.45 µM for the inactivated state under these experimental conditions.

### 3.6 Slow Inactivation

**FIGURE 7:**
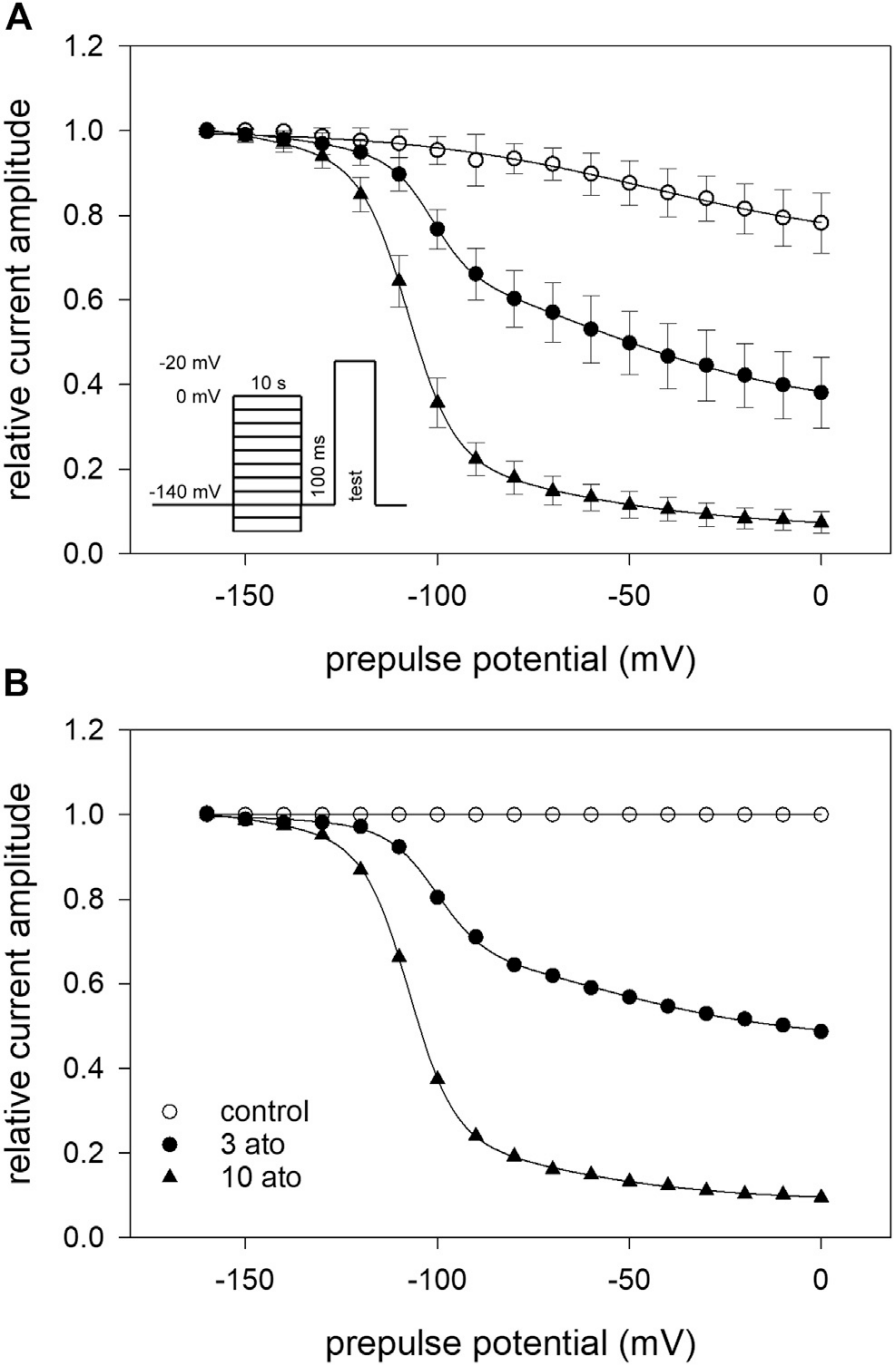
Interaction with the slow-inactivated state **(A)** Infrequent (interval: 30 s) channel activations by step depolarisations to −20 mV were elicited after long lasting (10 s) conditioning prepulses to different potentials (inset). A short (100 ms) recovery period at −160 mV was inserted immediately before the test pulse. Data of normalized peak currents are plotted vs. the prepulse potential. Solid lines are fits according to Eq. 5 for control (open squares) and atomoxetine (3 µM) (filled symbols). For fit parameters see Table 1. **(B)** Plot of renormalized data was obtained by dividing the drug values by control values. It is evident that the block also increases in the potential range (V50_2), were slow inactivation is expected to occur.

In a next set of experiments, we directly investigated for a possible interaction with the slow inactivated state. To this end, similar experiments as shown in Figure 4 were conducted with the following deviations: the duration of the conditioning prepulse was set to 10 s, and a recovery period (100 ms) at −160 mV was inserted immediately before the test pulse to eliminate or minimize the contribution from fast-inactivation. The experimental design is illustrated as inset in Figure 7A. The availability curve progressively decreased under control conditions with prepulse potentials positive to −100 mV. At the prepulse potential of 0 mV, 74.7 ± 3.2% of the channels were still available for activation. In the presence of atomoxetine (3 and 10 µM), the availability at 0 mV dropped to 33.4 ± 8.9% and 5.9 ± 0.8%, respectively (Figure 7A). Other fit parameters are listed in Table 1. The two most obvious changes here are the increase in the V_50_1_ component (a_1_), and the decrease in the residual current amplitude obtained at a prepulse potential of 0 mV. Another hallmark for an interaction with the slow inactivated state is an ongoing increase in inhibition in the potential range (V_50_2_) where slow inactivation is expected to occur. In order to evaluate this we renormalized the data in the presence of atomoxetine with their respective control (Figure 7B). The resulting potential-dependent behaviour suggests, that an interaction with the slow inactivation state is very likely to happen.

**TABLE 1:** Fit parameters for slow inactivation. Calculated fit parameters (Eq. 5) for the data of the interaction with the slow-inactivated state (*N* = 5).

	V50_1_ (mV)	k_1_ (mV)	a_1_ (%)	V50_2_ (mV)	k_2_ (mV)
*control*	−114.3 ± 8.0	10.8 ± 7.5	18.5 ± 15.9	−37.1 ± 5.1	23.2 ± 8.8
*ato (3 µM)*	−101.3 ± 0.3	5.2 ± 0.4	37.5 ± 18.7	−65.5 ± 2.8	31.8 ± 1.9
*ato (10 µM)*	−107.7 ± 0.2	5.9 ± 0.2	71.1 ± 19.7	−102.7 ± 3.9	34.2 ± 4.7

### 3.7 Recovery From Inactivation

**FIGURE 8:**
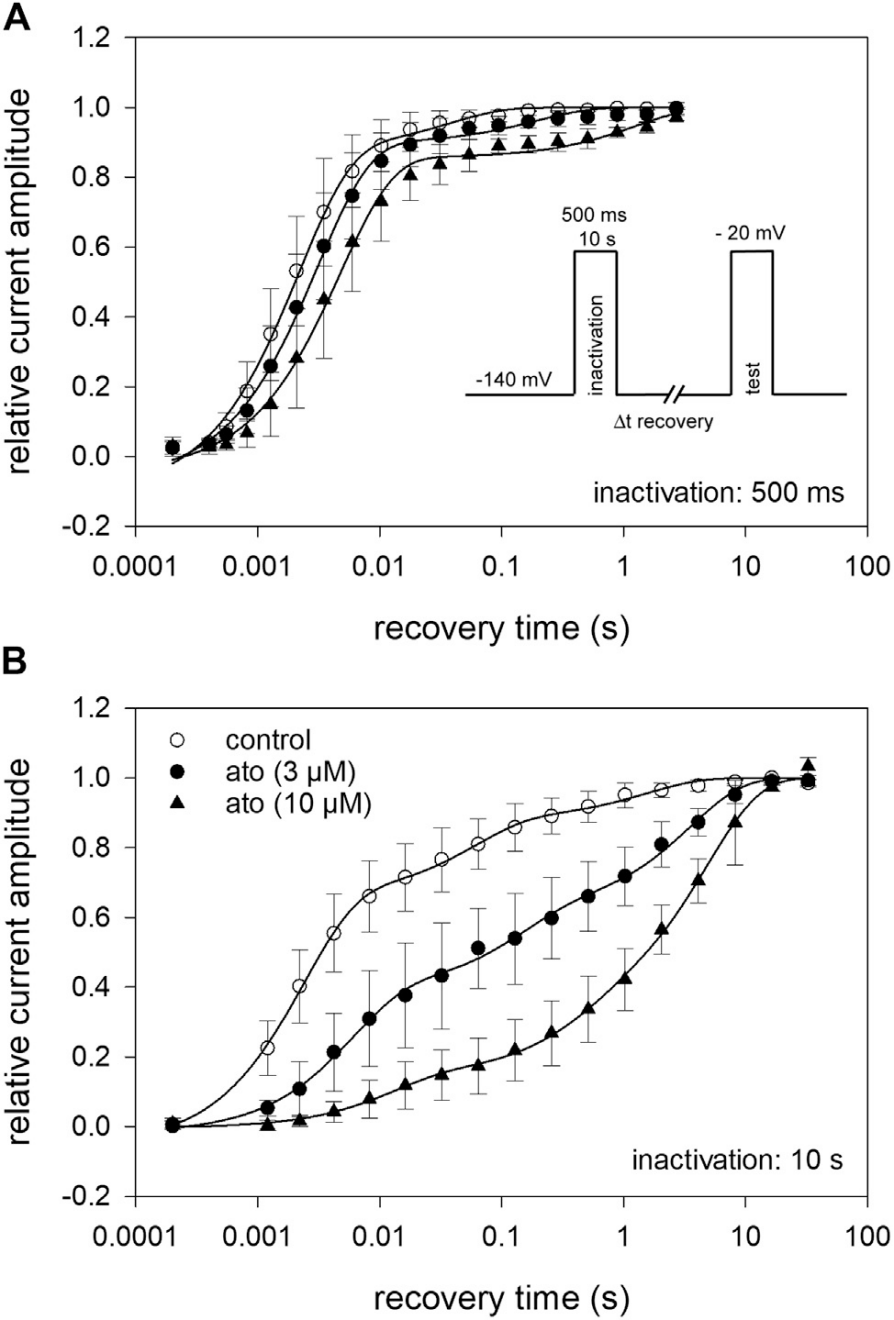
Recovery from inactivation **(A)** The relative amount of available channels after increasing duration of recovery at −140 mV is illustrated. Inactivation was performed at −20 mV for 500 ms. The pulse protocol is illustrated by the inset. Interval between individual measurements was 10 s and atomoxetine was applied at 3 and 10 µM. Solid lines represent fits with two exponential functions according to Eq. 10. **(B)** Otherwise identical protocol as in **(A)** but with the inactivation time set to 10 s. For data fitting, three exponentials were required. For fit parameters see Table 2, *N* = 5.

Recovery from inactivation was estimated with double pulse protocols using two different durations for the inactivating pulse: 500 ms and 10 s. The test pulse was applied after a variable time spent at the recovery potential of −140 mV (inset Figure 8). Using the short inactivation time of 500 ms, recovery occurred with two time constants. In the absence of atomoxetine about 90% of the channels recovered with a fast time constant (*τ*_1_ = 2.2 ± 0.2 ms), representing recovering from fast inactivation. The remainder recovered with a slower time constant (*τ*_2_ = 37.7 ± 15.6 ms), probably representing recovery from slow inactivation. In the presence of atomoxetine the time constant of the fast component was hardly extended whereas those of the slow time constant was prolongated with little or no change in the relative amount of the two components (Figure 8A, Table 2). These data indicate, that atomoxetine mainly impaired the process of slower component. Using an inactivation time of 10 s, recovery has to be fitted with triple exponential functions, separating the recovery process in a fast, intermediate and a slow component (Figure 8B). In the presence of atomoxetine a profound increase in all time constants resulted. Moreover, the component of the fast time constant decreased and that of the slow component increased in a concentration dependent manner while the amount of the intermediate component stayed almost constant (Table 3).

**TABLE 2:** Recovery from inactivation. Calculated fit parameters (Eq. 10) for the data of recovery from fast **(A)** and slow **(B)** inactivation (*N* = 5). **(A)** Inactivation: 500 ms

	*τ*_1_ (ms)	*τ*_2_ (ms)	*τ*_1_ (%)
*control*	2.2 ± 0.2	37.7 ± 15.6	88.9 ± 2.8
*ato (3 µM)*	3.1 ± 0.2	191.7 ± 74.2	90.3 ± 2.0
*ato (10 µM)*	4.7 ± 0.3	1,259 ± 351	86.2 ± 1.8

**TABLE 3:** **(B)** Inactivation: 10 s

	*τ*_1_ (ms)	*τ*_2_ (ms)	*τ*_3_ (ms)	*τ*_1_ (%)	*τ*_2_ (%)
*control*	2.34 ± 0.1	58.7 ± 11.5	1,551 ± 373	67.9 ± 1.7	20.8 ± 1.7
*ato (3 µM)*	5.8 ± 0.7	139 ± 37.2	3,524 ± 377	39.7 ± 2.2	22.7 ± 1.0
*ato (10 µM)*	12.4 ± 0.3	417 ± 152	4,859 ± 332	15.0 ± 1.6	17.2 ± 2.9

### 3.8 Use-dependent Interaction/Test for Open Channel Blocking Mechanism

**FIGURE 9:**
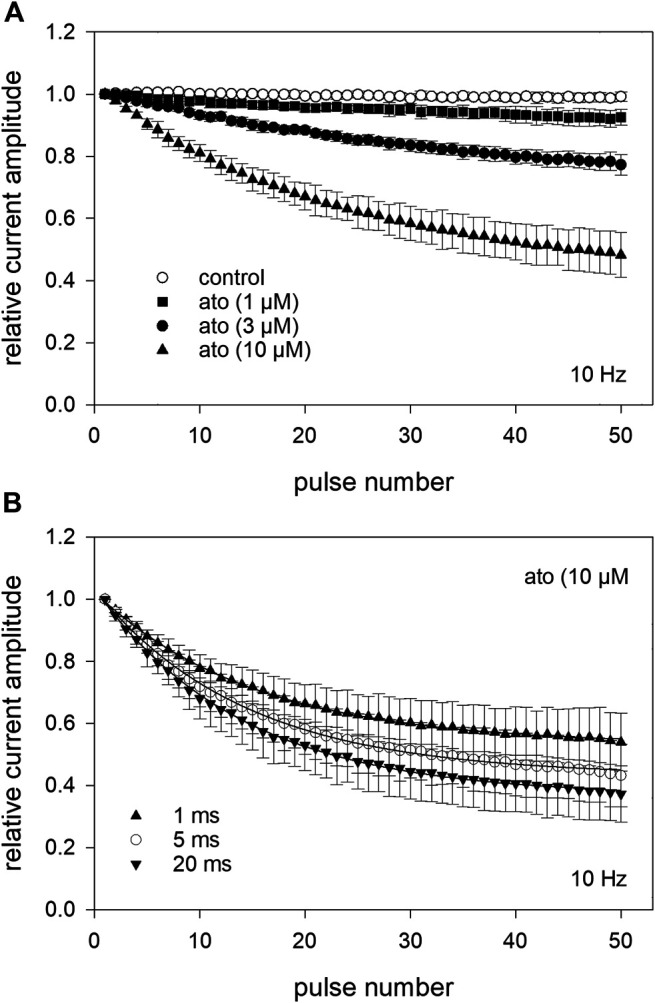
Analysis for use-dependence at a pulsing frequency of 10 Hz **(A)** After establishing stable control responses (activations every 5 s), atomoxetine was preincubated for 20 s before test pulses (1 ms) were applied at a frequency of 10 Hz. Under control condition (open circles), no use-dependence was obvious. In the presence of atomoxetine use-dependence increased with increasing concentrations of atomoxetine. The amplitudes which were obtained from the high frequency stimulation are related to the first pulse of each train. The relative current amplitudes of the 50th pulse were 0.93 ± 0.03, 0.79 ± 0.03, and 0.48 ± 0.07 for 1, 3, and 10 µM atomoxetine **(B)** Use-dependency slightly increases in the presence of 10 µM atomoxetine when the duration of the activation pulse is enlarged from 1 to 20 ms.

To investigate whether atomoxetine also interacts with the open channel, we first tested for a possible use-dependency. All these experiments were carried out at a holding potential of −140 mV and a test pulse to −20 mV with its duration set to 1 ms. This short activation time was chosen to minimize the time the channels spent in the inactivated state. After establishing stable baseline values using infrequent activations every 5 s (0.2 Hz) a train of high frequency stimulation (1, 3 10 Hz) was started. For evaluation, the peak current amplitude of each pulse was divided by the amplitude of the first response of the high frequency stimulation train. In the absence of atomoxetine, the channels revealed no use-dependent behaviour even at the highest frequency (10 Hz) (Figure 9A). When atomoxetine was preapplied for 20 s, a clear use-dependency could be observed. The extent of the current reduction strongly depended on the concentration of atomoxetine (Figure 9A) and the frequency with which the activations were carried out. For quantification, the remaining current amplitudes of the 50th pulse were related to the first pulse. At a stimulation frequency of 1 Hz, the relative current amplitudes dropped to 0.96 ± 0.01, 0.89 ± 0.05, and 0.69 ± 0.09 for 1, 3, and 10 µM atomoxetine, respectively (not shown in Figure 9). The corresponding relative current amplitudes were 0.95 ± 0.02, 0.85 ± 0.07, 0.61 ± 0.09 for 3 Hz (not shown in Figure 9), and 0,93 ± 0.03, 0.79 ± 0.03 and 0.48 ± 0.07, for 10 Hz, respectively (Figure 9A). In order to analyse further if atomoxetine prefers an interaction with the open or fast inactivated state, we increased the test pulse duration up to a factor of 20. It turned out, that the remaining current amplitudes dropped from 0.54 ± 0.03 to 0.43 ± 0.03 and 0.37 ± 0.09 when the activation time was increased from 1 to 5 or 20 ms (Figure 9B). As the increase in inhibition was small compared to the increase in activation/inactivation time, it can be assumed that the use-dependent block might arise from an interaction with the open state.

**FIGURE 10:**
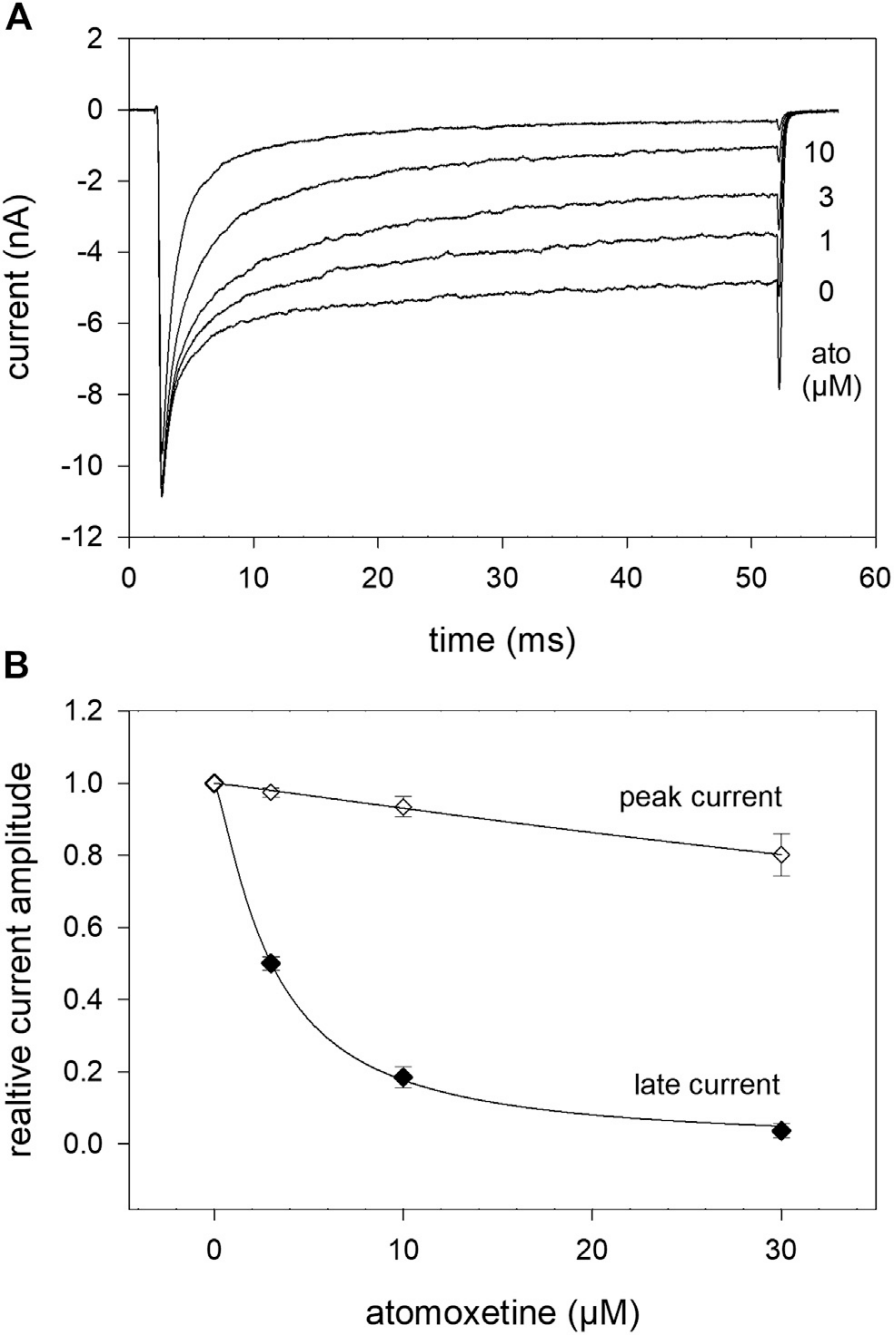
Interaction of atomoxetine with the open state. The possible interaction with the open state was analysed by means of the WCW mutant **(A)** Original current traces obtained in the absence and presence of different concentrations of atomoxetine (test pulse: 50 ms, −20 mV). Individual activations were carried out after a preincubation time of 30 s. Atomoxetine primarily diminished the late current measured at the end of the activation pulse. The peak current was less affected **(B)** Relative peak and plateau current amplitudes in relation to the concentration of atomoxetine.

In order to analyse the interaction with the open channel in more detail, we used inactivation-deficient mutant channels hNa_v_1.5_I408W_L409C_A410W (WCW mutant) with greatly reduced ability to inactivate. As can be seen from Figure 10, the peak current, representing drug binding to the resting state, was hardly affected in the presence of atomoxetine up to 10 µM. Due to this tiny effect on the peak current, it was not possible to state the half maximal effect here. By contrast, the late current was strongly diminished in a concentration dependent manner with half maximal inhibition occurring at 3.06 ± 0.4 µM atomoxetine (hill coefficient: 0.99 ± 0.17).

### 3.9 Binding Site

**FIGURE 11:**
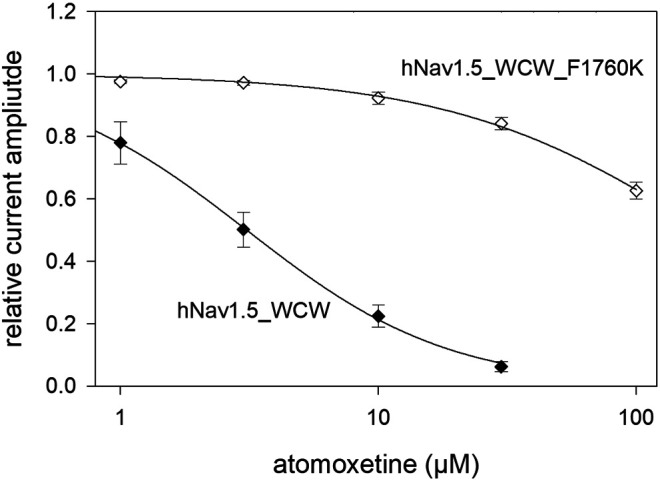
Possible interaction site for atomoxetine. Concentration-dependent current reduction of the late current of the WCW-mutant with a native and a modified anaesthetic binding site. The IC_50_ at WCW_F1760K mutant was 182 ± 11.9 µM while that of the unmodified WCW-mutant was 3.06 ± 0.4 µM. *N* = 5.

In a last set of experiments, we looked for a possible interaction site for atomoxetine with the hNa_v_1.5 channel. As many blockers of voltage-gated sodium channels operate via the binding site for local anaesthetics, we tested atomoxetine at the F1760K mutant which represents one out of several mutants which affects the interaction with local anaesthetics. To this end we engineered the F1760K mutant in the WCW background. It turned out that the affinity for atomoxetine was strongly reduced in the F1760K_WCW mutant compared to WCW alone (Figure 11). In particular, we estimated for the late current a half maximal effect of 182 ± 11.9 µM with a hill coefficient of 0.88 for the F1760K_WCW mutant. Compared to WCW mutant channels (IC_50_: 3.06 ± 0.4 µM), the half maximal effective concentration of atomoxetine is increased by more than 50-fold due to the inclusion of the F1760K mutation. Thus, a modification of the local anaesthetic binding site strongly affects the interaction of atomoxetine with the hNa_v_1.5 channel.

## 4 Discussion

Atomoxetine is primarily known as an inhibitor of the high affinity norepinephrine transporter system (Peterson et al., 2008). Here, we report that atomoxetine also operates as a potent blocker of VGSCs. It interacts with the human heart muscle sodium channel (hNa_v_1.5) in a state and use-dependent manner, with highest affinity for the inactivated and open state by interaction with the well-known binding site for local anaesthetics. The effective concentrations are within the therapeutic concentration range (Scherer et al., 2009; Ludolph et al., 2010).

As with many other drugs, the interaction of atomoxetine is not restricted to a single target. In fact, it interacts with many other diverse targets, like potassium channels (Scherer et al., 2009; Kobayashi et al., 2010) or ligand-gated ion channels such as TRPV1 (Gupta et al., 2014) and NMDA receptors, first described by our group and meanwhile confirmed by others (Ludolph et al., 2010; Di Miceli and Gronier, 2015; Barygin et al., 2017). However, even though fluoxetine and atomoxetine are very similar in their chemical structure, their mode of blocking the NMDA-receptor occurs by different mechanisms and different interaction sites (Barygin et al., 2017). As the related fluoxetine has previously been described to interact with VGSCs, an interaction of atomoxetine with this type of channel was not unexpected (Lenkey et al., 2006; Poulin et al., 2014). Thus, after having confirmed the sodium channel blocking activity of atomoxetine, we were mainly interested to elucidate the blocking mechanism.

Meanwhile, there exist several reports about the sodium channel blocking activity of fluoxetine. The two most important thereof, which present an entire investigation in detail, were conducted at hippocampal cell cultures and stable transfected hNa_v_1.5 channels (Lenkey et al., 2006; Poulin et al., 2014). As there are large differences by which mechanism fluoxetine interacts with either the neuronal or heart muscle sodium channel (Lenkey et al., 2006; Poulin et al., 2014), we mainly focused on our comparison to data obtained from heart muscle channels.

### 4.1 State-dependent Interaction

Fluoxetine and atomoxetine have in common that the interaction with the hNa_v_1.5 channel strongly depends on the membrane potential. Comparing the affinity of fluoxetine at a holding potential of −140 mV (resting state) with that of a holding potential of −90 mV (about resting membrane potential of heart muscle cells), the ratio was less than 10-fold (Poulin et al., 2014). We did not operate with fixed membrane potentials in the range where inactivation occurs, as the amount of inactivated channels is not fixed for a given membrane potential. Moreover, the amount of inactivated channels increases in a time-dependent manner. Therefore, we referred our data to the inactivated state by considering the current amount of inactivated channels. In this way, we found, depending on the applied protocol, an about 50-fold higher affinity for the inactivated state compared to the resting state. In any case, the resultant ratio, which is consistently used as a measure for the “safety-margin” for a drug, is much higher for atomoxetine than for fluoxetine (Poulin et al., 2014). Assuming that the resting membrane potential of heart muscle cells is around −90 mV, our data obtained for slow inactivation (Figure 7) would indicate, that the inhibitory effect of atomoxetine is already nearly fully developed at this membrane potential. However, the experimental data cannot be directly transferred to the *in vivo* situation. One critical point is, that the potential dependency monitored here might be shifted by 15–20 mV in negative direction. Such a shift is generally observed when fluoride containing internal solutions are used (Jarecki et al., 2008). We and many others use fluoride as a main anion in the internal solution as such solutions allow stable measurement for long time. Thus, if inactivation occurs *in vivo* at more positive membrane potentials, the inhibitory effect of atomoxetine would be much less at the resting membrane potential. Another important role comparing the *in vitro* with the *in vivo* situation play other cellular components such as the presence or absence of different auxiliary ß-subunits. In this way, a right shift in the inactivation is observed for the TTX resistant Na_v_1.8 channel in the presence of the ß1 subunit (Leffler et al., 2007). As atomoxetine preferentially interacts with the inactivated state of the hNa_v_1.5, the knowledge of the real percentage of channels which are at rest in the inactivated state is a prerequisite to estimate the impact which atomoxetine exerts *in vivo* at rest.

### 4.2 Interaction With Fast/Slow Inactivated State

An important question regarding the interaction with the inactivated state is whether this occurs via the fast- or the slow-inactivated state or by both states. Fluoxetine clearly interacts with the fast-inactivated state. This is taken from the left shift of the inactivation curve (inactivation time 400 ms) as well as from the prominent delay in recovery even after very short inactivation pulses (40 ms) (Poulin et al., 2014). Atomoxetine behaved different with no shift in the inactivation curve and only a minor delay in recovery when the inactivation time was fixed to 500 ms. From the block development experiments, it is evident that prominent interactions occur not until inactivation time lasts longer than 1 s. Consequentially, atomoxetine exerted stronger effects in protocols with more long-lasting inactivation pulses (10 s). This holds for the delay in the recovery experiments as well as for the potential dependent behaviour analysing slow inactivation. Regarding the recovery from slow inactivation it would be speculative, whether the fast and intermediate component correlate to the recovery of drug-free and/or drug-modified channels. However, the strongly prolonged time constant in combination with the increased amount of the slowly recovering channels might arise from a slow dissociation of atomoxetine from inactivated channels. A similar behaviour was also observed for bupivacaine (c.f. (Schulze et al., 2014)). The potential dependent behaviour after prolonged inactivation also revealed strong drug effects. Even here, a clear correlation to an interaction with either the fast or slow inactivated state cannot be provided. Because two Boltzmann functions were required for appropriate data fitting, several parameters resulted. The most obvious changes were observed for the relative amount of each function and the residual current amplitudes at a prepulse potential of 0 mV. Another observation is that individual curves revealed two different slopes, probably reflecting two different processes and/or states. In this way, the function with the more positive midpoint exhibiting a shallow slope might represent slow inactivation whereas the function with the more negative midpoint showing a steep slope might represent fast or intermediate inactivation (Wang and Wang, 2014). If slow inactivation would be restricted to the part with the more positive midpoint, it would contribute to about one half of the total inhibition in case of 3 µM atomoxetine. The remainder could represent slow interaction with a fast or an intermediate state. Alternatively, total inhibition must be ascribed to slow inactivation as also midpoints for slow inactivation shift to more negative values in the presence of local anaesthetics (Lenkey et al., 2006). Nonetheless, the different slopes remain unexplained. A similar strong change in the slope has previously been reported upon the application of mexiletine or lidocaine to Na_v_1.5 but not so when applied to Na_v_1.7 channels (Wang et al., 2015). A further hint that the part with the more positive midpoint might represent slow inactivation arises from data normalization with respect to control. It is evident that the inhibition increases in a potential range where slow inactivation is expected to occur. So far, it is impossible to discriminate between an interaction with the slow inactivated state from a slow interaction with the fast-inactivated state (Karoly et al., 2010). Hence, there are currently also no hints about a different physiological outcome when a drug interacts in either way with an ion channel.

### 4.3 Interaction With the Open State

For many diseases such as epilepsy or arrhythmia, an interaction with open state upon high frequency stimulation offers favourable properties as regular sodium channel activity would be less affected or even unaffected. Both drugs, fluoxetine and atomoxetine, reveal a strong use-dependency. However, the time course of inhibition is different. In the presence of fluoxetine, an almost stable value is reported to occur after about 10 activations using a pulse duration of 10 ms and stimulation frequency of 10 Hz (Poulin et al., 2014). For atomoxetine, no stable plateau value was obtained even after 50 activations. The reason for this discrepancy does not reside in the short activation time (1 ms) which we used in the present work, as even with 20 ms lasting pulses no stable plateau value was achieved (Figure 9B).

### 4.4 Interaction With Channel Mutants

The use of channel mutants is a favored method to elucidate the mode of interaction of a drug with an ion channel. Two of them are very prominent: WCW (I408W_L409C_A410W) and F1760K (Wang et al., 2003; Wang and Wang, 2014; using L407W_L409C_A410W). For the WCW mutant, fast inactivation is strongly reduced whereas in case of the F1760C mutant, an interaction with the local anaesthetic binding site is affected. In case of the WCW-mutant, the ratio for the interaction with the resting state (peak current) vs. open channel (late current) was 3-fold for fluoxetine (Poulin et al., 2014). For atomoxetine, no such quotient could be calculated as the interaction with the resting state was too low to estimate an IC_50_ value, indicating a high ratio value. In case of the anaesthetic binding site (F1760), the ratio in affinity between F1760C and wildtype was about 2-fold for fluoxetine. We tested the F1760K-mutant in the WCW background and found an about 50-fold reduced affinity for the mutant. Thus, the two related drugs also strongly differ in this regard. Concerning the time-dependent interaction, there was also a marked difference between wild-type and WCW mutant channels. Using 3 µM atomoxetine, a time constant of about 10 s was required for wild-type channels to achieve a steady state condition, whereas with the WCW mutant, this occurred in less than 10 ms.

### 4.5 Clinical Implications

Atomoxetine reveals two important properties with respect to sodium channels of heart muscle type: use-dependency and slow interaction. Use-dependency could diminish the transient peak current amplitudes giving rise to a pro-arrhythmic behaviour. By contrast, the slow interaction is expected to reduce the late current whereby it would exert an anti-arrhythmic effect (Belardinelli et al., 2013). This raises the question about the predominance of either effect under normal or pathological conditions. Our data clearly indicate that atomoxetine had little or no effects at very negative membrane potentials. However, the resting membrane potential of ventricular cardiac cells is around −90 mV so that depending on the applied dose a very small or a dramatic block of hNa_v_1.5 channel could occur. In case of a prominent impact this could affect the action potential threshold, the upstroke velocity and the early repolarization phase leading to life-threatening situations, similarly as seen in patients with *SCN5A* loss-of-function mutations, like Brugada syndrome or cardiac conduction disease (Zimmer and Surber, 2008). So far, no severe cardiac side effects are reported which are based on the administration of atomoxetine to our knowledge. However, rhythm disturbances without clinical significance or a discontinuation of medication treatment due to any cardiovascular effect are known from rare cases (Tanidir et al., 2015; Hennissen et al., 2017). On the other hand, the late current mimicked by the non-inactivating WCW mutant was reduced to about 50% when the same concentration of atomoxetine was applied. This suggests that the drug has an anti-arrhythmic potential. The effect should be minimal in ADHD patients, because wild-type hNa_v_1.5 channels inactivate nearly completely within a few milliseconds. Atomoxetine treatment, however, could be interesting for carriers of genetic mutations associated with long QT syndrome (LQT). A hallmark of LQT3 is an inactivation defect in hNa_v_1.5 mutant channels, leading to an increased persistent current, and consequently, to prolonged action potentials and to higher susceptibility for cardiac events (Bennett et al., 1995). A specific reduction of this persistent current fraction, as observed in our study with the WCW mutation, could qualify atomoxetine as an anti-arrhythmic drug in respective monogenetic cardiac disorders.

As state-dependent sodium channel blockers reveal a poor subtype selectivity, it can be expected that atomoxetine also affects other sodium channels including those occurring in neurons. Of note, the data obtained here for sodium channel of the heart muscle are in closer accordance with data for fluoxetine obtained from neurons vs. those from sodium channels of heart muscle type (Ilyin et al., 2005; Lenkey et al., 2006; Poulin et al., 2014). Moreover, as in neurons much higher activation frequencies are likely to occur, use-dependency is here of greater importance than at heart muscle cells. Therefore, it is not unlikely that atomoxetine at clinically relevant concentrations even diminishes excessive neuronal activity, thereby being potentially anti-convulsive.

All in all, it should be considered that data from experimental settings, as provided here, cannot directly by transferred to the clinical situation since temperature and many other parameters might be different between these two settings (Wang et al., 2004; El-Battrawy et al., 2016).

## 5 Conclusion

Atomoxetine blocks hNa_v_1.5 channels in a state- and use-state-dependent manner. These effects occur well within the clinically relevant concentration range. Concerning its possible impact on heart muscle sodium channels, it reveals stronger anti-arrhythmic than pro-arrhythmic properties.

## Data Availability

The raw data supporting the conclusions of this article will be made available by the authors, without undue reservation.
